# Freedom, Justice, and Neglected Tropical Diseases

**DOI:** 10.1371/journal.pntd.0001235

**Published:** 2011-08-30

**Authors:** Carlos Franco-Paredes, Jose I. Santos-Preciado

**Affiliations:** 1 Hospital Infantil de Mexico, Federico Gomez, Mexico, D.F., Mexico, and Rollins School of Public Health, Emory University, Atlanta, Georgia, United States of America; 2 Unidad de Medicina Experimental, Facultad de Medicina, Universidad Nacional Autonoma de Mexico, Mexico, D.F., Mexico

*“What moves us, reasonably enough, is not the realization that the world falls short of being completely just—which few of us expect—but that there are clearly remediable injustices around us which we want to eliminate”* [1]

Neglected tropical diseases (NTDs) are remediable injustices of our times. Poverty is the starting point, and the ultimate outcome, of NTDs. Much about poverty is evident enough, but considering poverty as simply low income is insufficient [2]. In the context of NTDs, poverty should be seen as the relative deprivation of freedoms and capabilities dictating a lack of opportunities and choices in life [3], [4]. Capabilities refer to the person's freedom to lead one type of life or another, and freedom with the real opportunity to accomplish what we value as human beings [5]. Thus, NTDs are diseases of socially excluded populations that promote poverty by relatively depriving individuals from basic capabilities and freedoms. The social pathways of becoming ill with an NTD include socially determined failures including widespread illiteracy, malnutrition, poor living conditions, unemployment, and the overall failure of ownership relations in the form of entitlements [2], [5]. In turn, in a vicious cycle of destitution and dispossession, NTDs produce disability, disfigurement, stigma, and premature mortality.

When addressing issues surrounding social equity and justice it becomes inescapable to revisit the writings of Amartya Sen, one of the world's leading intellectuals of our time. Throughout his books, but most emphatically in his recent one, *The Idea of Justice*, he argues for a framework for the critical assessment of judgments about justice whether based on *freedoms*, *capabilities*, *resources*, or *well-being* [1]. According to Sen, liberty is defined as the possible fields of application of equality, and equality as the pattern of distribution of liberty; and living may be seen as a set of interrelated functionings, consisting of beings and doings [5]. A person's achievements in this respect can be seen as the vector of constitutive functionings, including elementary ones such as being adequately nourished and being in good health [4], [5]. The health status of an individual in a specific social arrangement can be scrutinized from two different perspectives: the actual achievement of health, and the freedom to achieve it [3]. Health achievement tends to be a reliable guide to the underlying capabilities of an individual and a central consideration of human life. At the same time, the freedoms and capabilities that we are able to exercise in our lives are dependent on our health achievement [3], [5]. Taking into account the role of health in human life, social justice calls for a fair distribution as well as efficient creation of human capabilities and opportunities for individuals to achieve and maintain good health [3]. Therefore, achieving health free from escapable or preventable illness, disability, and premature death, which occurs with most NTDs, is an integral component of justice in our lives that is directly influenced by our existing freedoms and capabilities [6].

As human beings, our ability to manage our food supply around 10,000 years ago set in motion a chain of social and cultural development that propelled us into the globalized modern world [7]. Social arrangements of human populations transformed: political systems and economic structures developed; hierarchies, power relations, and social inequalities along with population growth emerged, switching the balance of freedoms and choices. These transitions have produced some benefits, but also some penalties, in the health status of the world's population [7].

Indeed, throughout the history of humankind, suffering illnesses has governed our lives. Despite our sophisticated human design, vulnerability to disease is an inevitable part of our developmental origin as individuals and of our evolutionary origin as species [8]. Our biological susceptibility is defined by a myriad of ancestral molecular compromises and trade-offs acquired during our biological history as a human species through ecological clashes with environmental factors [9]. Beyond our biological predisposition, there are social processes that influence the occurrence of illnesses [10]. Regretfully, there is consistent evidence that socially disadvantaged populations have poorer survival chances and premature death due to socially determined diseases than more socially favored groups [11]. No law in nature decrees that individuals die from diseases that are preventable and treatable such as occurs with most NTDs.

In a seminal paper in 1992, Margaret Whitehead defined inequity in health as the occurrence of health differences considered unnecessary, avoidable, unfair, and unjust, thus adding a moral and ethical dimension to health inequalities [12]. Health equity does not refer only to the fairness in the distribution of health or the provision of health care; rather, it is linked with the larger issues of fairness and justice in social arrangements [4]. In this regard, many individuals positioned at the bottom of the social ladder find themselves living a life with few choices and few opportunities to avoid becoming ill, receive treatment, and prevent the long-term disability and premature death associated with most NTDs.

Some notions of nominal political freedoms can be applied to the violation of freedoms leading to negative health outcomes associated with NTDs [13]. However, other destructive social arrangements place individuals at a risk of becoming ill with an NTD, such as inability to satisfy hunger or achieve sufficient nutrition; lack of freedom to obtain treatments for NTDs; insufficient opportunities to be adequately clothed or sheltered; gender inequalities; unavailability of clean water or sanitary facilities to prevent the acquisition of an NTD; the lack of public facilities and social care including health care and educational facilities; or effective institutions for maintenance of order and peace [4], [7]. Health inequities associated with NTDs systematically place populations at further social disadvantage [14]. For example, in Sri Lanka, lymphatic filariasis is a leading cause of deformity, disfigurement, permanent physical disability, and stigma that promotes social isolation, emotional distress, and delayed diagnosis treatment [15]. These factors, in turn, promote loss of productivity and income, which pushes this category of patients with low visibility and their households from extreme poverty to destitution. The life they lead is thus restricted and escaping the trap of poverty becomes distant.

Similar to the example of lymphatic filariasis in Sri Lanka, a large number of the world's population suffers from NTDs that stem from inadequate social arrangements. These arrangements also impact the distribution of health resources, producing inequalities in the distribution of health care for those ill with an NTD or suffering from the long-term complications of an NTD (social policies linked to wealth, power, and prestige; and social hierarchies that deprive people of the opportunity for receiving or utilizing health resources, the allocation of health care resources, financing of health care; and quality of health care services) [3]. In many settings, NTDs affecting indigenous populations represent a historical legacy of social injustices. An example is the impact of the African slave trade in the spread of NTDs in Latin America and the Caribbean leading to a significant burden of disease. In this region, many indigenous populations are disenfranchised and poor, and thus NTDs have been largely forgotten diseases even though their collective disease burden exceeds that of HIV/AIDS, tuberculosis, or malaria [16].

The health of people around the world is a global responsibility, and the right to the highest attainable standard of health goes far beyond health care to promote social entitlements and freedoms [17]. Preventing, treating, and rehabilitating those at risk of or suffering from NTDs will promote people's capabilities and opportunities and return a sense of dignity and self-realization into their lives. In this sense, targeting NTDs in a comprehensive fashion represents a clear and feasible poverty alleviation strategy that ultimately fosters social equity. Reducing the burden of NTDs is a grand social intervention to promote social change, advance justice, and increase freedom of marginalized populations. Fairness, as the prospect of mutually advantageous cooperation among equal members of the human species [18], is a trait that we should endure. Human suffering stemming from NTDs needs to cease being one of the shadows that delineate social inequity, injustice, and a biological destiny of poverty.

## References

[1] Sen A (2009). The idea of justice.

[2] Sen A (1981). Poverty and famines. An essay on entitlement and deprivation.

[3] Sen A (2002). Why health equity?. Health Econ.

[4] Sen A (2000). Development as freedom.

[5] Sen A (1995). Inequality reexamined.

[6] Sen A, Farmer P (2003). Pathologies of power.. Health, human rights, and the new war on the poor.

[7] Weiss RA, McMichael AJ (2004). Social and environmental risk factors in the emergence of infectious diseases.. Nat Med.

[8] Nesse RM, Stearns SC (2008). The great opportunity: evolutionary applications to medicine and public health.. Evol App.

[9] McCallum CJ (2007). Does medicine without evolution makes sense?. PLoS Biol.

[10] Marmot M (2006). Health in an unequal world.. Lancet.

[11] Gruskin S, Braveman P (2003). Defining equity in health.. J Epidemiol Community Health.

[12] Whitehead M (1992). The concepts and principles of equity and health.. Int J Health Serv.

[13] Beyrer C, Villar JC, Suwanvanichkij V, Snigh S, Baral SD (2007). Neglected diseases, civil conflicts, and the right to health.. Lancet.

[14] Franco-Paredes C, Jones D, Rodriguez-Morales AJ, Santos-Preciado JI (2007). Improving the health of neglected populations in Latin America.. BMC Public Health.

[15] Perera M, Whitehead M, Molyneux D, Weerasooriya M, Gunatilleke G (2007). Neglected patients with a neglected disease? A qualitative study of lymphatic filariasis.. PLoS Neglect Trop Dis.

[16] Hotez PJ, Bottazzi ME, Franco-Paredes C, Ault SK, Roses-Periago M (2008). The neglected tropical diseases of Latin America and the Caribbean: estimated disease burden and distribution and a roadmap for control and elimination.. PLoS Negl Trop Dis.

[17] Hunt P (2006). The human right to the highest attainable standard of health: new opportunities and challenges.. Trans R Soc Trop Med Hyg.

[18] De Waal F (2008). Primates and philosophers. How morality evolved.

